# Native reading direction influences lateral biases in the perception of shape from shading

**DOI:** 10.1080/1357650X.2014.990975

**Published:** 2014-12-24

**Authors:** Austen K. Smith, Izabela Szelest, Trista E. Friedrich, Lorin J. Elias

**Affiliations:** ^a^ Department of Psychology, University of Saskatchewan, Saskatoon, SK, Canada

**Keywords:** Eye tracking, Visual attention, Pseudoneglect, Lighting bias, Target finding

## Abstract

Although neurologically normal individuals often exhibit leftward biases of perception and attention, known as pseudoneglect, factors such as lighting, spatial location and native reading direction have been found to modulate these biases. To investigate lighting and spatial biases in left-to-right and right-to-left readers search times were measured in a target finding task where lighting and target locations were manipulated. Target search times under upper-left lighting were significantly shorter than lower-left, upper-right and lower-right lighting among left-to-right readers. Right-to-left readers did not display the same leftward bias, even displaying significantly shorter search times under upper-right lighting than those of left-to-right readers. Significantly shorter search times for targets located in the upper-left quadrant (compared to other quadrants) were observed for left-to-right readers, while search times for upper-right located targets were significantly shorter for right-to-left readers compared to those of left-to-right readers. Participant scan times of stimuli divided into equal quadrants were monitored by an eye-tracking camera. Both groups displayed greater scan times in upper quadrants. These findings suggest that native reading direction modulates spatial and light perception biases resulting in weaker leftward, or a lack of lateral biases among right-to-left readers.

Due to the complexity and quantity of information that enters the human eye, the visual system has learned how to increase processing efficiency by selectively attending to certain elements and making assumptions to produce seemingly simple percepts. Ambiguously lit stimuli are processed by the human visual system using prior assumptions about the location of the light source as well as about the shape and form of the object—and what the end result of these two interacting factors should be (McManus, Buckman, & Woolley, 2004; Ramachandran, 1988). Two-dimensional (2D) images are perceived as three-dimensional (3D) using shading and lighting cues, more commonly referred to as perceiving shape from shading. Manipulating the shading of 2D images has proven to be an effective method in determining assumptions about light source perception made by the human visual system (Ramachandran, 1988; Sun & Perona, 1998). A circle shaded on a vertical gradient, i.e. shaded darker at the top than the bottom (and vice versa), looks to be a convex ball or concave bowl (Figure 1). As real-world shading is most often from overhead light sources, consistent with a vertical gradient shading, this 3D sphere illusion disappears when lighting is applied from the sides. The widely accepted explanation of human visual system optimization for perceiving light from above is our existence on a planet with a single overhead light source, the sun (Kleffner & Ramachandran, 1992; Ramachandran, 1988).

**Figure 1.**
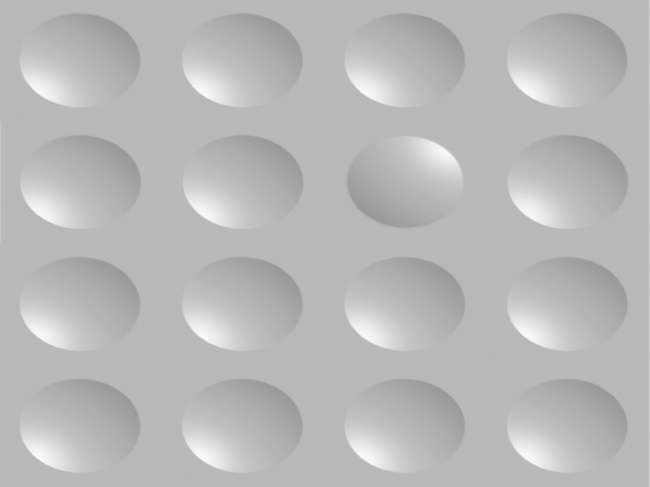
A target finding task was implemented using stimuli devoid of 3D cues other than lighting and shading, creating targets of either convex balls or concave bowls. Note that the stimuli, as in previous work from our laboratory, are ellipsoids rather than spheres.

Applying shape from shading to a target finding paradigm, Sun and Perona (1998) and McManus et al. (2004) observed faster target identification times when the field appeared to be lit from the upper-left. Using a similar method with stimuli consisting of either one sphere or mirror-reversed pairs, one on top of the other, Elias and Robinson (2005) also observed a leftward lighting bias. As well, Mamassian and Goutcher (2001) found that shape interpretation was biased on a shape discrimination task when lighting was from the upper-left. Unlike the aforementioned preference for light from above, the leftward lighting bias seems to have no intuitive explanation.

Scientific explorations into lighting asymmetries and leftward lighting biases have been fuelled by German Gestalt psychologist Wolfgang Metzger's early observation of left light and right light not being perceptually equivalent (as in Sun & Perona, 1998). Portraits, photographs and advertisements are more commonly lit from the left, than from the right (Labar, 1973; McManus et al., 2004; Thomas, Burkitt, Patrick, & Elias, 2008), and when given the choice, individuals prefer locating the light at an angle left of the vertical when illuminating paintings (McDine, Livingston, Thomas, & Elias, 2011). To increase realism in paintings and portraits, artists commonly add shading to a scene that is consistent with other surrounding elements to match how the observer would expect to experience the scene in real life. Sun and Perona (1998) suggest that upper-left lighting might have higher perceptual value, as they report the light source in 225 master paintings, across varied schools and periods, to be predominately located in the upper-left.

In addition to the leftward lighting bias, asymmetries of perception in neurologically normal individuals occur in line bisection (McCourt, 2001), image comparisons (Nicholls, Bradshaw, & Mattingley, 1999) and aesthetics (Chokron & De Agostini, 2000). The underlying cause of these biases, as well as *if* there is indeed a shared underlying mechanism, is debated. This persistent leftward bias is known as pseudoneglect (Bowers & Heilman, 1980; Nicholls et al., 1999) and is thought to result from hemispheric asymmetries, specifically the dominance of the right parietal lobe in spatial processing (Corbetta, Shulman, Miezin, & Petersen, 1995; Posner & Petersen, 1990). However, a common trend throughout the behavioural sciences is to collect data from undergraduate students at universities in Western, educated, industrialized, rich and democratic societies (Henrich, Heine, & Norenzayan, 2010), with few researchers accounting for disparities that may exist between different cultural groups with opposite directions of reading. Scanning habits resulting from reading direction has arisen as a potential explanation influencing spatial asymmetries. When tasks typically eliciting pseudoneglect are employed with populations whose native languages read and write in directions other than left-to-right, results have been mixed (Chokron & Imbert, 1993; Rinaldi, Di Luca, Henik, & Girelli, 2014). Leftward perceptual biases could to a degree result from a sample of individuals whose visual explorations mirror reading and writing text from left-to-right.

By accounting for cultural differences of participants, in particular for their native reading direction, leftward biases of perception, attention and aesthetics have been more clearly understood (Chokron & De Agostini, 2000; Fagard & Dahmen, 2003; Rinaldi et al., 2014). Findings from studies examining scanning habits suggest that learning a language that reads right-to-left, rather than left-to-right, modulates performance on visual spatial tasks. Rinaldi et al. (2014) examined Italian monolingual left-to-right readers, Israeli monolingual right-to-left readers and Israeli bilinguals performance on an adapted star cancellation task and a line bisection task. Monolingual left-to-right readers made more omissions on the cancellation task on the right side than on the left and made more right side omissions than monolingual right-to-left readers. Bilinguals showed no difference in omissions, while monolingual right-to-left readers made more leftward omissions, but not significantly so. When completing the cancellation task monolingual left-to-right readers made more left-to-right horizontal shifts while monolingual right-to-left readers made more right-to-left horizontal shifts. In line bisections, regardless of line length (80 mm or 160 mm), monolingual left-to-right readers displayed a consistent leftward bias while monolingual right-to-left readers showed a rightward bias. Rinaldi et al. found the direction of a language system to modulate visual spatial task performance and posit an *Interactive Account* (cultural and biological factors) rather than a pure neurobiological account of spatial asymmetries.

Scanning strategies used to arrive at a fixation point differ between Western, Middle Eastern and East Asian groups, which suggest that scanning patterns used during examination of non-directional stimuli are influenced by native reading direction (Abed, 1991). Abed found individuals who read and write English to make more left-to-right eye movements when examining non-directional scenes, whereas right-to-left readers, oppositely and to a lesser degree made more right-to-left eye movements. Similarly, Morikawa and McBeath (1992) suggest that eye movements associated with learning to read a language are responsible for differences observed between left-to-right and right-to-left readers when presented with several still images in rapid succession, creating the illusion of motion. Left-to-right readers displayed a significant leftward motion bias while right-to-left readers displayed no bias. Of direct relevance to the current study, the first analysis of the influence of native reading direction on light source perception using eye tracking to measure attention found differentiation between left-to-right and right-to-left readers in a forced choice paradigm that used left and right-lit images. Left-to-right readers significantly preferred left-lit images and fixated significantly more on the left side of images, whereas right-to-left readers (non-significantly) preferred right-lit images and fixated more (non-significantly) on the right side (Smith & Elias, 2013).

A general explanation for visual spatial differences between groups with opposite reading directions has yet to emerge. Several studies have not found an effect of scanning habits (Ishii, Okubo, Nicholls, & Imai, 2011; Nicholls & Roberts, 2002) and dispute the influence of cultural factors on leftward spatial biases. The leftward lighting bias has not been scrutinized for cultural variability of participants in the same way other tasks have, like line bisection (Chokron & Imbert, 1993), grey scales (Nicholls & Roberts, 2002), reading (Nazir, Ben-Boutayab, Decoppet, Deutsch, & Frost 2004; Pollatsek, Bolozky, Well, & Rayner, 1981) and aesthetic preference (Chokron & De Agostini, 2000; Ishii et al., 2011). However, a recent study by Andrews, Aisenberg, D'Avossa, and Sapir (2013) observed a significantly smaller leftward lighting bias among Hebrew participants, compared with English participants, on a shape from shading task using honeycomb stimuli. With the exception of Andrews et al. (2013) and the aforementioned study by Smith and Elias (2013), few investigations concerned with the potential impact of scanning habits—influenced by reading direction—on the leftward lighting bias phenomenon have been carried out.

We used a target finding task made up of stimuli devoid of 3D cues other than lighting and shading, creating targets of either convex balls or concave bowls (Figure 1). The direction of light illuminating the stimuli and the location of the target sphere were manipulated with resulting search times measured. Concurrently, eye movements were monitored and the scan time of the four equal sections of stimuli was measured. We investigated: (1) lighting conditions leading to shortest target search times, (2) target locations leading to shortest target search times and (3) quadrant scanning time. This third measure did not provide any information about the amount of time taken to search for a target, but rather allocations of scan time. Our predictions mirror the rationale of an Interactive Account, proposed by Rinaldi et al. (2014), in that both cultural factors and neurobiological factors will determine the outcomes of these three investigations. We predict that a pre-existing left visual field advantage (driven by right parietal dominance for spatial processing) in neurologically normal left-to-right reading individuals, in conjunction with learned scanning habits favouring leftward space, will result in an overall strong leftward bias. Scanning habits of right-to-left readers will favour rightward space, balancing out the innate left visual field advantage, resulting in an overall rightward bias or at minimum a weak leftward bias. Upper-left lighting conditions are predicted to lead to shortest target search times for left-to-right readers while shortest target search times are expected to occur under upper-right lighting conditions for right-to-left readers. Furthermore, left-to-right readers are predicted to exhibit shorter search times for upper-left located targets and right-to-left readers are predicted to display shorter target search times for upper-right located targets. Lastly, we predict that left-to-right readers will demonstrate increased scan times in the upper-left quadrant, while right-to-left readers' scan times will be greatest in the upper-right quadrant.

## METHOD

The Research Ethics Board at the University of Saskatchewan approved this study.

### Participants

Sixty-eight left-to-right reading University of Saskatchewan undergraduate students received course credit for participation. Data were not collected from eight participants due to eye-tracking difficulty (glasses, dark eye make-up) or experimenter error. In two instances, participant eye movements were recorded for less than half of all trials and did not meet minimum criteria for usability. The remaining 58 (18 male) left-to-right reading participants had an average age of 20.2 (*SD* = 3.6) and were right-handed (3 left-handed).

Thirty-two right-to-left reading individuals participated in the study. Data were not collected from one participant due to eye-tracking difficulties. Of the remaining 31 participants, 3 individuals were left-handed, 12 were female and the average age was 30.2 (*SD* = 5.9). Seventeen individuals had previously participated in research studies in our lab, but all were naïve to the goals of this task. All participants were bilingual, with their time spent in Canada varying from a few months to a few years. Participants received remuneration for their time.

### Materials

A SensoMotoric Instruments (SMI) Remote Eye-Tracking Device (RED II) recorded participant eye movements at 60 Hz. Eye movement data were processed by iView (3.1) software running on a custom built workstation by SMI. Presentation of stimuli was on a 1024 × 768 resolution 19-inch LCD display by E-prime (v.1.2) running on a separate computer, linked by serial connection to the SMI workstation. A chin rest, approximately 700 mm from the computer display, was used to help reduce movement. Stimuli were images of a 4 × 4 array of shaded spheres with one target sphere, loosely based on other target finding experiments using illuminated spheres (McManus et al., 2004; Sun & Perona, 1998). Each image consisted of 15 spheres with the same lighting angle and one odd one out with an opposite lighting angle, e.g. 15 spheres at +45° and one at –135° (Figure 1). Spheres could be illuminated from 16 different angles, 0°, ±22.5°, ±45°, ±67.5°, ±90°, ±112.5°, ±135°, ±157.5° or 180° (negative leftward) with the target in 1 of 16 locations, resulting in 256 test trials. To reduce overall testing time for right-to-left readers stimuli with spheres lit from 180° and 0° lighting angles were excluded, resulting in 224 test trials. To be consistent, these trials were also excluded from data analysis of left-to-right readers. Test trials were randomized and preceded by a calibration exercise and practice trials.

Newer, but comparable, hardware and software were purchased midway through the study and used for data collection of right-to-left reading participants. The new eye-tracking camera (RED 4) was built by the same company (SMI) and recorded at the same frequency. Experiment Center 3.0 (SMI) and a higher resolution display (with images displayed at a resolution consistent with those presented to left-to-right readers) were used for stimuli presentation. Handedness and footedness effects were accounted for as potential covariates using the Waterloo Footedness Questionnaire–Revised developed by Elias, Bryden, and Bulman-Fleming (1998). Basic demographics, including first language and occurrences of sinistral relatives were collected in the questionnaire.

### Procedure

Participants were welcomed and seated at a desk in a small windowless room with overhead fluorescent lighting. A brief explanation of the consent form and the questionnaire (Elias et al., 1998) was given, followed by time to ask questions and complete the forms. After informed consent was obtained, participants positioned themselves comfortably in the chair and chin rest so they were centred to the computer display. Verbal and on-screen instructions were given (in English for all participants) to search for the odd sphere out and press the space bar as soon as it was found. Following each trial, a fixation cross appeared for 1000 msec. Thirty-four participants completed only one block of trials (due to time constraints), while 55 participants completed two blocks, with a short rest in-between. In this later case an average of the two blocks was analyzed. Before leaving, participants were thanked for their participation and given a debriefing form explaining the experiment and rationale.

### Coding and analysis

Eye tracking data were converted to a plain text format with data in each cell indicating the amount of time a quadrant was visually explored, where each row denoted trials and columns corresponded to each of the four quadrants. Trials (rows) were grouped by the condition under which they occurred: light from the upper-left, lower-left, upper-right or lower-right in combination with upper-left, lower-left, upper-right or lower-right located targets. Like trials were aggregated and data were structured with conditions ranging from upper-left lighting/upper-left located targets to lower-right lighting/lower-right located targets.

## RESULTS

A repeated-measures analysis of variance (ANOVA) was carried out to test the relationships of within-subjects variables lighting direction (four levels: upper-left, lower-left, upper-right and lower-right light) and target location (four levels: upper-left, lower-left, upper-right and lower-right located targets) and between-subjects variable reading direction (two levels: left-to-right and right-to-left). Main effects of lighting direction, *F*(3, 261) = 22.25, *p* < 0.001, 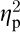 = 0.204, and target location, *F*(3, 261) = 10.05, *p* < 0.001, *η* ^2^_p_ = 0.104, were observed. There was no significant main effect of reading direction, *F*(1, 87) = 2.94, *p* = 0.09, 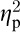 = 0.033. A significant interaction was observed between reading direction and lighting direction, *F*(3, 261) = 20.57, *p* < 0.001, *η* ^2^_p_ = 0.191, while the interaction between reading direction and target location approached significance, *F*(3, 261) = 2.49, *p* = 0.06, 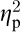 = 0.028. Significant interactions between lighting direction and target location, *F*(9, 783) = 3.19, *p* = 0.001, *η* ^2^_p_ = 0.035, and lighting direction, target location and reading direction, *F*(9, 783) = 6.88, *p* < 0.001, 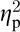 = 0.073, were observed.

The main effects of lighting direction and target location were not predicted and as such a corrected alpha of 0.015 was used in post hoc comparisons. It was determined that target identification was significantly quicker when the field was illuminated with upper-left lighting rather than lower-left, upper-right or lower-right lighting, *t*(88) = 3.97 *p* < 0.001, *t*(88) = 6.41, *p* < 0.001, *t*(88) = 8.63, *p* < 0.001, respectively. Target identification times for upper-left located targets were significantly quicker than lower-left located targets, *t*(88) = 4.76, *p* < 0.001, upper-right located targets, *t*(88) = 4.63, *p* < 0.001 and lower-right located targets, *t*(88) = 4.44, *p* < 0.001.

**Figure 2.**
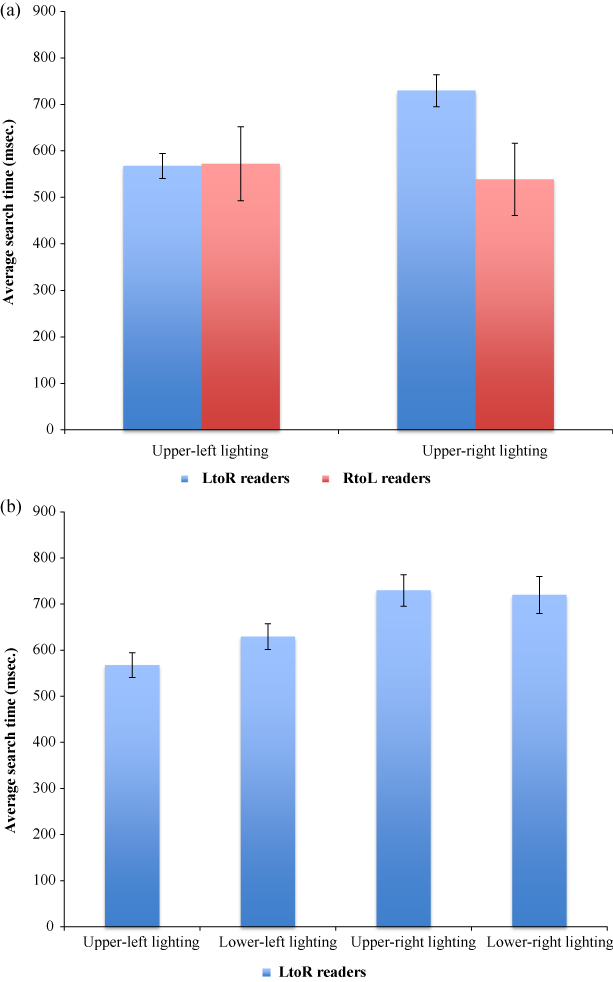
(a) When the field was illuminated from the upper-right, average target search times for right-to-left readers were significantly less than left-to-right readers. Error bars represent 95% confidence intervals; and (b) In the left-to-right reading group target search times were significantly shorter when the field was illuminated from the upper-left. Error bars represent 95% confidence intervals.

The interaction between reading direction and lighting direction was examined with independent samples *t*-tests comparing target identification times under upper-left lighting and under upper-right lighting of left-to-right readers with right-to-left readers (a priori predictions). Left-to-right readers were not significantly quicker at identifying targets under upper-left lighting, *t*(87) = 0.136, *p* = 0.45 (one-tailed) than right-to-left readers. Under upper-right lighting, however, right-to-left readers were significantly quicker at identifying targets than left-to-right readers, *t*(87) = 5.08, *p* < 0.001 (one-tailed; Figure 2a). Within the left-to-right reading group, paired samples *t*-tests found that target identification under upper-left lighting was significantly quicker than target identification under lower-left, upper-right and lower-right lighting, *t*(57) = 6.62, *p* < 0.001, *t*(57) = 13.24, *p* < 0.001, *t*(57) = 11.76, *p* < 0.001, respectively (all one-tailed; Figure 2b). Within the right-to-left reading group, paired samples *t*-tests revealed that target identification was significantly quicker under upper-right lighting when compared to lower-left and lower-right lighting, *t*(30) = 2.763, *p* = 0.005 and *t*(30) = 3.55, *p* = 0.0005 (one-tailed) and approached significance when compared to upper-left lighting, *t*(30) = 1.601, *p* = 0.06 (one-tailed).

**Figure 3.**
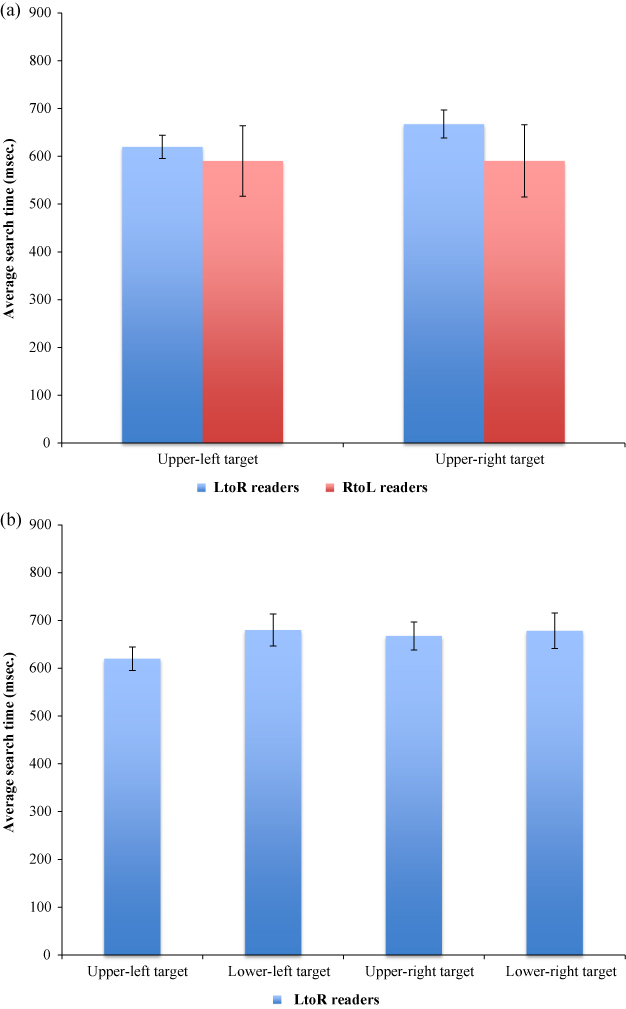
(a) Right-to-left readers identified upper-right located targets significantly quicker than left-to-right readers. Error bars represent 95% confidence intervals; and (b) In the left-to-right reading group average search times were significantly shorter for targets located in the upper-left. Error bars represent 95% confidence intervals.

Given the clear trend towards significance (*p* = 0.06) the interaction between reading direction and target location deserves further examination. Independent samples *t*-tests comparing target identification times for upper-left and upper-right located targets of left-to-right readers with right-to-left readers (a priori predictions) show that there was no difference between groups for upper-left located targets, *t*(87) = 0.92, *p* = 1.81, but that right-to-left readers identified upper-right located targets significantly faster than left-to-right readers, *t*(87) = 2.21, *p* = 0.015 (both one-tailed; Figure 3a). Within left-to-right readers, upper-left located targets were identified significantly quicker than lower-left, upper-right and lower-right located targets, *t*(57) = 5.59, *p* < 0.001, *t*(57) = 6.03, *p* < 0.001, *t*(57) = 4.76, *p* < 0.001, respectively (all one-tailed; Figure 3b). Upper-right located targets were not identified significantly quicker than upper-left, lower-left or lower-right targets among right-to-left readers (*t*(30) = 0.02, *p* = 0.492, *t*(30) = 1.28, *p* = 0.105, *t*(30) = 0.02, *p* = 0.100, respectively; all one-tailed).

An ANOVA testing the relationship between within-subjects variable quadrant scanning time (four levels: scanning time in upper-left, lower-left, upper-right and lower-right quadrants) and between-subjects variable reading direction (two levels: left-to-right and right-to-left) found a significant main effect of quadrant scanning time, *F*(3, 261) = 8.41, *p* < 0.001, 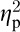
= 0.088, and a significant interaction, *F*(3, 261) = 7.30, *p* < 0.001, 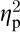 = 0.077. The main effect of reading direction was not significant, *F*(1, 87) = 2.47, *p* = 0.120, 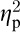
= 0.028.

Post hoc comparisons examining the main effect of quadrant scan time used a corrected alpha of 0.015, as it was not predicted. Mean scan time of the upper-right quadrant was greater than other quadrants, with significant differences between upper-right and lower-left scan times, *t*(88) = 3.71, *p* < 0.001, between upper-right and lower-right scan times, *t*(88) = 4.99, *p* < 0.001, but not between upper-right and upper-left scan times (*t*(88) = 0.81, *p* = 0.419).

**Figure 4.**
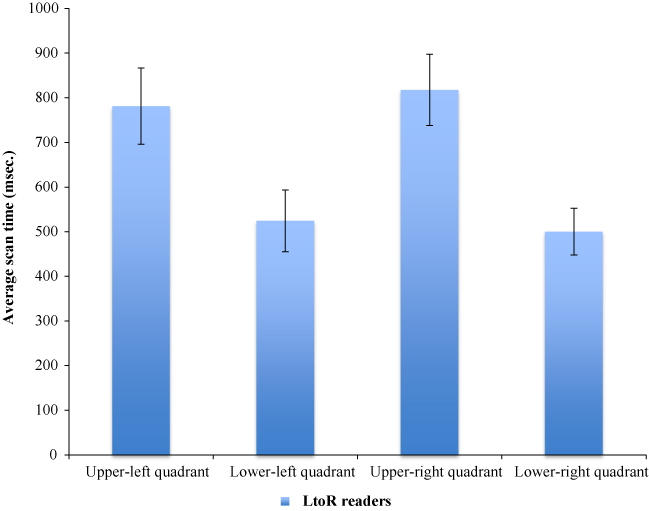
Left-to-right readers' upper-left quadrant average scan times were significantly greater than lower-left and lower-right quadrants, but not upper-right. Error bars represent 95% confidence intervals.

The interaction between quadrant scan time and reading direction was further investigated using independent samples *t*-tests comparing scan times of upper-left and upper-right quadrants of left-to-right and right-to-left readers (a priori predictions). Left-to-right readers spent more time scanning both upper-left quadrants, *t*(87) = 2.63, *p* = 0.005 (one-tailed) and (not predicted) upper-right quadrants, *t*(87) = 2.93, *p* = 0.004, than right-to-left readers. However, as left-to-right readers spent more time overall exploring each quadrant, of greater interest, perhaps, are differences between each quadrant within each group. In planned paired samples *t*-tests left-to-right readers spent significantly more time scanning the upper-left quadrant than the lower-left quadrant, *t*(57) = 4.30, *p* < 0.001, and lower-right quadrant, *t*(57) = 4.82, *p* < 0.001, but not the upper-right quadrant (*t*(57) = –0.68, *p* = 0.251; all one-tailed; Figure 4). Right-to-left readers, however, did not significantly scan one quadrant more than others, *t*(30) = 0.50, *p* = 0.309, *t*(30) = 0.10, *p* = 0.461, *t*(30) = 0.56, *p* = 0.29, respectively; all one-tailed.

## DISCUSSION

Previously it has been reported that neurologically normal individuals misperceive objects in the left hemifield as brighter, more numerous and larger than those in the right hemifield, even if they are equivalent (Nicholls et al., 1999). Further, quicker target identification has been observed when a scene appears to be lit from the upper-left (McManus et al., 2004; Sun & Perona, 1998). This leftward attentional bias is commonly referred to as pseudoneglect (Bowers & Heilman, 1980) and although the effects of pseudoneglect have been demonstrated across a wide array of tasks, little attention has been paid to the possible influence that native reading direction could have. A disproportionate amount of studies have used samples of undergraduate students in Western societies, with their findings being generalized to explain behaviours of all human beings. Pertaining to visual perception and spatial reasoning this assumption has been found to be flawed, as Western, educated, industrialized, rich and democratic societies are among the least representative (Henrich et al., 2010). We used a target finding task comprised of stimuli devoid of 3D cues other than lighting and shading, creating targets of either convex balls or concave bowls. Consistent with past studies, we observed quicker target identification times among left-to-right readers when the scene was illuminated with upper-left lighting, compared to other lighting directions. However, by using a sample of right-to-left readers, results differed. Compared to left-to-right readers, right-to-left readers identified targets quicker in scenes illuminated by upper-right light. A similar pattern seen in the lighting direction results is observed in the target spatial location results. Left-to-right readers identified upper-left located targets quicker than targets in other locations and right-to-left readers identified upper-right located targets quicker than left-to-right readers. Based on these results we propose that the cultural influence of reading and writing alters perception and suggest that there is more to the story when it comes to perceptual asymmetries of light sources, and perhaps even pseudoneglect, than was once thought.

The first language an individual learns appears to influence spatial attention (Chokron & Imbert, 1993; Fagard & Dahmen, 2003), and might be related to differences in the direction of eye movements (Abed, 1991; Smith & Elias, 2013). Behaviours of right-to-left readers observed in the current study follow trends of weaker leftward, or a lack of lateral, biases observed in studies employing different cognitive tasks while accounting for native reading direction (Andrews et al., 2013; Morikawa & McBeath, 1992; Rinaldi et al., 2014). In the few studies that have considered native reading direction, findings of leftward biases and pseudoneglect effects are often not observed, highlighting the importance of ascertaining a culturally diverse sample when investigating perceptual asymmetries (Henrich et al., 2010). Some recent studies have found perceptual differences between left-to-right and right-to-left readers (Andrews et al., 2013; Rinaldi et al., 2014; Smith & Elias, 2013) and posit that directional scanning (influenced by reading habits) and neurobiological factors (hemispheric dominance for spatial processing) concomitantly modulate spatial biases. The current study is unique in that in conjunction with a shape from shading task participants were eye tracked in an attempt to ascertain an additional indirect measure of attention. Although lateral differences in eye-tracking results between groups were not particularly revealing it is clear that quadrants in the upper visual field were explored more as both groups displayed greater scan times in the upper-left and upper-right quadrants. The absence of any statistically significant lateral biases in either group in eye-tracking data in the current study is perplexing and, as discussed below, requires further investigation to determine if these results are in error or if indeed lateral eye-movement effects are small.

Regardless of native reading direction group or target spatial location, quicker target identifications occurred when the field of spheres was lit from above (either from the left or the right). These conditions lead to a target appearing as a concave bowl among a field of convex ball distractors—consistent with Kleffner and Ramachandran's (1992) seminal finding that concave targets are easier to identify against backgrounds of convex distractors. The current study contributes new information to the literature about lateral lighting biases between groups. In line with predictions, left-to-right readers identified targets quickest when the scene was illuminated from the upper-left and right-to-left readers displayed a lack of bias for leftward lighting, even producing results approaching significance (*p* = 0.06) for a rightward lighting bias. Further, right-to-left readers identified targets quicker than left-to-right readers when the scene was illuminated from the upper-right. Regarding target spatial location, quickest target identification occurred for both groups when targets were located in upper quadrants. Following from our predictions, left-to-right readers found targets located in the upper-left quicker than targets located anywhere else while right-to-left readers identified upper-right located targets quicker than their left-to-right reading counterparts. Overall scan times in quadrants were similar in both groups, as upper quadrants were explored more than lower quadrants. Predictions made for lateral biases in scan time for each group are still unclear given the data from the current study and require further investigation. It is difficult to speculate on the degree to which real-world perception is affected, but we propose that biases of light source perception and target identification depend on the direction one's native language is read and written.

The samples used in the current study are a limitation to our findings. As most left-to-right reading participants were English monolingual, ideally the study would be designed to match them to monolingual right-to-left readers. Given our geographical location in the Canadian plains, obtaining this sample was not feasible; our sample consisted of participants who spoke English and at least one right-to-left reading language. Further, better documentation of exact amounts of time spent reading and writing a left-to-right language and/or living in a culture that is predominately left-to-right reading, is an opportunity for future research. Another possible limitation of our findings lies in the design of our test trials. Past target finding studies have used *catch trials*, or trials where no target is present, to ensure the participant is accurately completing the task. Although we did not employ catch trials, we did an additional analysis of scan time allocation and target spatial location which yielded significant results suggesting that when a target was located in a certain quadrant, the scan time of that quadrant was typically greater than the others—implying that the task was done correctly.

More studies collecting eye-tracking data during visual spatial tasks from both left-to-right and right-to-left readers are needed. The greater allocation of time spent scanning the upper-left and upper-right quadrants in the current study is intriguing, however, the lack of lateral biases of scan time in both groups could be explored in future studies with a greater focus on visual exploration. More studies, covering a broader range of perceptual tasks, are needed before the mechanism responsible for the differences observed in the present study can be confidently identified. We propose that the intense training one undergoes in learning to read and write either works with or against left visual field advantages, driven by right parietal dominance for spatial processing, not unlike the Interactive Account of visual spatial asymmetries, as proposed by Rinaldi et al. (2014). In the case of learning a left-to-right language, an individual is trained to focus attention first to the left and then make their way to the right, and then back to the left, enabling a leftward bias already in place by neurological organization. For an individual who is learning to read and write a right-to-left language, attention is directed in an opposite manner with scans beginning on the right and working left and then returning to the right. Attention is preferentially directed to the right visual field, of which the right parietal lobe normally receives (processes) less information from, compared to the left visual field. This may have a neutralizing effect on leftward biases—either by increasing the spatial ability of the left parietal lobe, or by decreasing practice of the right parietal lobe.
